# Tislelizumab Plus Chemotherapy Sequential Neoadjuvant Therapy for Non-cCR Patients After Neoadjuvant Chemoradiotherapy in Locally Advanced Esophageal Squamous Cell Carcinoma (ETNT): An Exploratory Study

**DOI:** 10.3389/fimmu.2022.853922

**Published:** 2022-06-02

**Authors:** Wenwu He, Chenghao Wang, Lei Wu, Gang Wan, Baisen Li, Yongtao Han, Haojun Li, Xuefeng Leng, Kunyi Du, Haijun Chen, Qifeng Wang, Lin Peng

**Affiliations:** ^1^ Department of Thoracic Surgery, Sichuan Cancer Hospital & Institute, Sichuan Cancer Center, School of Medicine, University of Electronic Science and Technology of China, Chengdu, China; ^2^ Department of Radiotherapy, Sichuan Cancer Hospital & Institute, Sichuan Cancer Center, School of Medicine, University of Electronic Science and Technology of China, Chengdu, China; ^3^ Department of Medical Affairs, BeiGene (Beijing) Co., Ltd., Beijing, China

**Keywords:** neoadjuvant chemoradiotherapy, neoadjuvant chemoimmunotherapy, total neoadjuvant therapy, esophageal squamous cell carcinoma, tislelizumab, protocol

## Abstract

**Background:**

Esophageal squamous cell carcinoma (ESCC) remains a challenging malignant tumor with poor prognosis and limited treatment methods worldwide, and most patients are at a locally advanced stage at diagnosis. High recurrence and metastasis rates remain the main factors leading to the failure of the current standard treatment of neoadjuvant chemoradiotherapy plus surgery for resectable locally advanced ESCC. Improving the pathological complete response (pCR) rate may significantly benefit the survival of patients with resectable locally advanced ESCC after neoadjuvant therapy.

**Methods:**

Tislelizumab plus sequential neoadjuvant chemotherapy was administered to non-clinical complete response (cCR) patients after neoadjuvant chemoradiotherapy for locally advanced ESCC. The patients then received surgery and adjuvant therapy according to the postoperative pathological results. Eighty patients with locally advanced ESCC were recruited for the study. The primary outcomes of the pCR rate and the incidence of adverse events will be analyzed completely within 24 months, and the secondary endpoints will include cCR rate, major pathological response rate, objective response rate, R0 resection rate, event-free survival, and overall survival.

**Discussion:**

This study explored the safety and efficacy of tislelizumab plus chemotherapy sequential neoadjuvant therapy for non-cCR patients and provided a total neoadjuvant therapy model that can benefit patients with locally advanced ESCC.

**Clinical Trial Registration:**

ClinicalTrials. gov NCT05189730. Registered: November 26, 2021, https://register.clinicaltrials.gov/prs/app/action/SelectProtocol?sid=S000BBD5&selectaction=Edit&uid=U0004UG3&ts=47&cx=e0cm59.

## Introduction

Esophageal cancer was the fifth leading cause of cancer-related deaths worldwide in 2018 (1). However, in China, it is fourth leading cause of cancer-related deaths in 2015 (2). Esophageal squamous cell carcinoma (ESCC) is the most common histological subtype of esophageal cancer (3). The annual incidence of ESCC in China is approximately 53% of that worldwide (2). In China, ESCC is a common malignant tumor with regional aggregation and high mortality; the patients are usually at the locally advanced stage at diagnosis.

Based on the CROSS (4) and NEOCRTEC5010 (5) clinical trials, the NCCN guidelines recommend neoadjuvant chemoradiotherapy followed by surgery for locally advanced ESCC. However, local recurrence and distant metastasis after neoadjuvant chemotherapy remain the main causes of mortality (6, 7). According to the latest outcome of the NEOCRTEC5010 study, patients with postoperative pathological complete response (pCR) after neoadjuvant chemoradiotherapy had longer survival than patients without pCR (8). Therefore, currently, improving the pCR rate in postoperative patients after neoadjuvant chemoradiotherapy and reducing recurrence and metastasis have become the most important aspects of ESCC research.

In recent years, immunotherapy has shown expected results in advanced esophageal cancer (9–12), and has been used for neoadjuvant therapy (13). Another important study showed that the total neoadjuvant therapy (TNT) model significantly increased the odds of pCR in neoadjuvant therapy for colorectal cancer (14). Therefore, we initiated this phase-II clinical trial to explore the role of TNT in ESCC. We believe that this study will improve the pCR rate of postoperative patients after neoadjuvant therapy and reduce recurrence and metastasis, which is different from previous studies, and will be a revolutionary study on ESCC.

## Methods

### Statement of Ethics Approval

This study was approved by the ethics committee of Sichuan Cancer Hospital, China. The ethics certificate number is SCCHEC-02-2021-056. All the included patients signed informed consent forms. The trial will be conducted according to the ethical principles of the Declaration of Helsinki (15).

### Study Design and Setting

**Figure 1:**
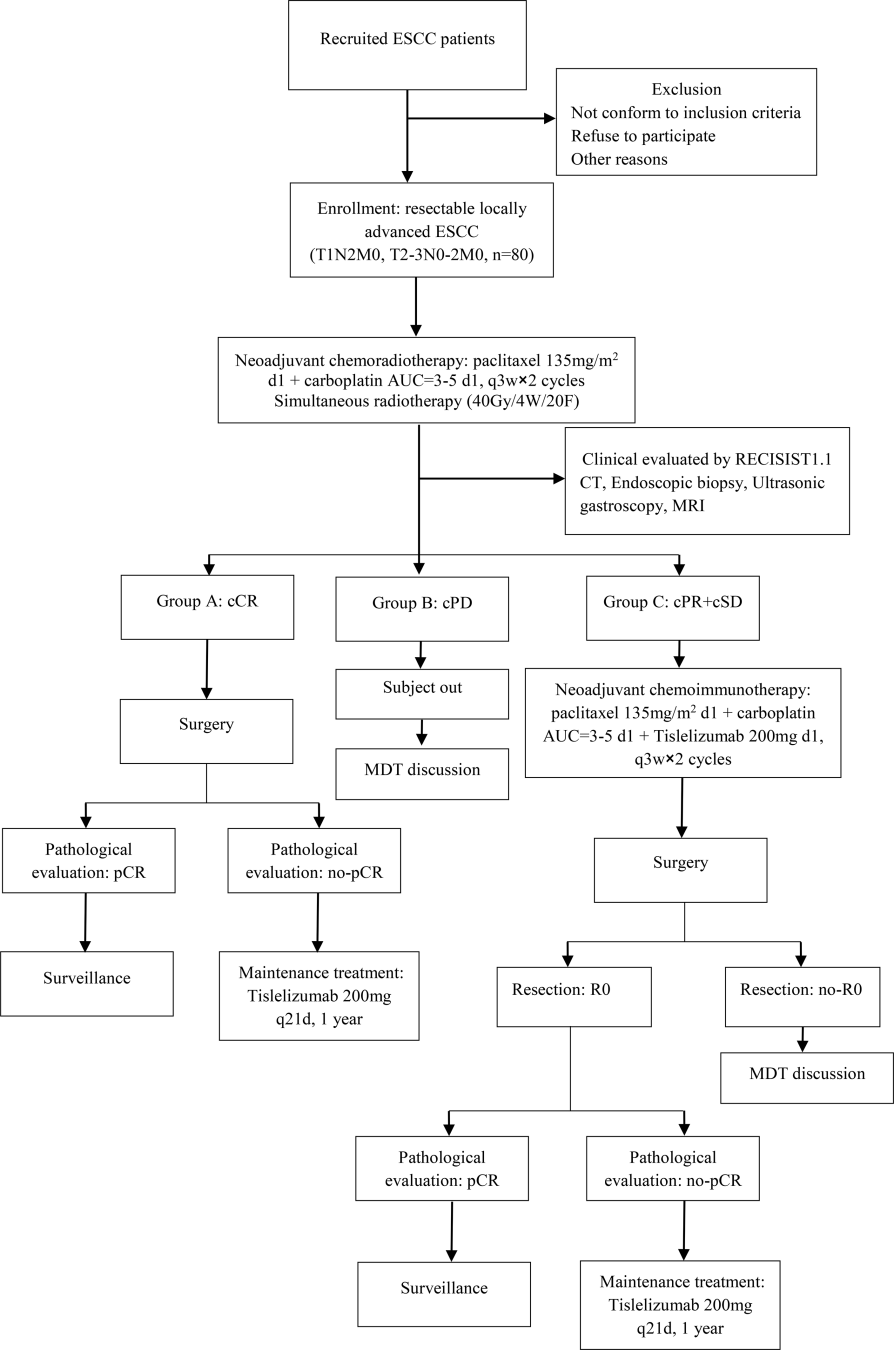
Flowchart of the exploratory study. ESCC, esophageal squamous cell carcinoma; cCR, clinical complete response; cPD, clinical progressive disease; cPR, clinical partial response; cSD, clinical stable disease; MDT, multidisciplinary team; pCR, pathology complete response.

This was a phase II, single-center, open-label clinical trial. Eligible patients received neoadjuvant chemoradiotherapy [paclitaxel 135 mg/m^2^ d1 + carboplatin AUC = 3–5 d1, q3w × 2 cycles + simultaneous radiotherapy (40 Gy/4 W/20F)] treatment. Then, the efficacy was evaluated according to RECIST 1.1. Patients with a clinical complete response (cCR) undergo surgery after 4–6 weeks. After surgery, patients with pCR would always undergo surveillance, and patients with non-pCR would receive immunotherapy (Tislelizumab 200 mg q21d) and maintenance treatment for one year. If patients were evaluated for clinical progressive disease (cPD), they would receive a new treatment regimen after a multidisciplinary team (MDT) discussion. Other patients with clinical partial response (cPR) and clinically stable disease (cSD) received two cycles of neoadjuvant immunochemotherapy (paclitaxel 135 mg/m2 d1 + carboplatin AUC = 3–5 d1 + Tislelizumab 200 mg d1, q3w × 2 cycles). Subsequently, all the patients underwent surgery after 4–6 weeks. After surgery, the patients with R0 resection were divided into two groups: patients with pCR who would always undergo surveillance and patients with non-pCR who would receive immunotherapy (Tislelizumab 200 mg q21d) maintenance treatment for one year. The other patients who did not undergo R0 resection received a new treatment regimen after MDT (**Figure 1**). This study aimed to investigate the safety and effectiveness of the TNT model for treating locally advanced ESCC.

In addition, cCR patients must conform to the following criteria: 4 weeks after the end of radiotherapy and chemotherapy, the MDT team was required to jointly determine whether the patient had achieved clinical complete remission through enhanced computed tomography (CT), gastroscopic biopsy, bronchoscopy, ultrasound endoscopy, cervical color ultrasound, and magnetic resonance imaging (MRI).

Firstly, the contrast-enhanced CT scan showed that the esophageal tumor had completely regressed. Second, no tumor cells were found on gastroscopic biopsy, and thoracic MRI showed that the wall of the esophagus was normal.

### Study Endpoints

The primary outcome was the pCR rate, defined as no evidence of residual tumor cells in the primary site and resected lymph nodes of the operative specimens. The most important objective was to analyze the pCR rate in the whole non-cCR/cPD population who underwent sequential chemotherapy combined with immunotherapy after neoadjuvant chemoradiotherapy. The incidence of adverse events (AEs) was defined as the proportion of participants who experience treatment-related AEs as assessed by National Cancer Institute Common Terminology Criteria for Adverse Event, Version 4.0 (CTCAE v4.0). In subsequent research, we will also assess the consistency of cCR and pCR.

Secondary outcomes included cCR rate, major pathological response (MPR) rate, objective response rate (ORR), R0 resection rate, events free survival (EFS), and overall survival (OS).

The time range for reporting AEs in each participant will be up to three months after the last dose of neoadjuvant treatment. MPR is defined as the residual tumor after neoadjuvant treatment of ≤ 10% residual tumor lesions in surgical specimens. EFS was defined as the time from the date of inclusion to the date of the first documented non-fatal event (worsening cardiac function, hospitalization for congestive heart failure, liver function impairment, liver cirrhosis, transformation to AML, as defined in the protocol) or death, whichever occurred first. OS was defined as the duration from disease diagnosis to death for any reason.

### Participants

Patients with ESCC who were diagnosed without any prior treatment in Sichuan Cancer Hospital underwent physical examination; gastroscopy; ultrasonic gastroscopy; contrast-enhanced CT scan of the neck, chest, and abdomen; cervical and abdominal ultrasound; esophagography; electrocardiography; and pulmonary function tests. If the patient agreed, positron emission tomography-CT(PET/CT) was performed. All patients will be given pretreatment clinical staging according to the 8th UICC for International Cancer Control TNM system (16). Written informed consent was obtained from all patients before they were recruited.

### Inclusion Criteria

(1) Histologically confirmed locally advanced resectable thoracic ESCC.
(2) Clinical stage T1-4aN+ and T3-4aN0 based on the AJCC8-TNM system
(3) Age between 18 and 75 years
(4) At least one measurable lesion in accordance with RECIST 1.1.
(5) Eastern Cooperative Oncology Group (ECOG) performance status of 0–1;
(6) The expected survival time was > 6 months.
(7) No diagnosis of other cancers and no prior anticancer therapy history.
(8) There were no operative contraindications.
(9) The important organs functions meet the following requirements: the absolute neutrophil count (ANC) ≥ 1.5 × 10^9^/L; the platelet count ≥ 100 × 10^9^/L; hemoglobin ≥ 90 g/L; bilirubin less than or equal to 1.5 times ULN; ALT and AST less than or equal 2.5 times UILN; creatinine clearance rate (CCr) ≥ 50 mL/min; normal thyroid function
(10) Female subjects of childbearing potential having a negative pregnancy test result and agreeing to take effective contraceptive measures during the study period and within 3 months after the last dose.
(11) Be willing and able to provide written informed consent/assent for the trial.

### Exclusion Criteria

(1) Confirmed patients with distant metastasis
(2) Infectious diseases requiring treatment
(3) Active autoimmune disease or documented autoimmune disease or symptoms requiring systemic hormone or anti-autoimmune drug treatment
(4) Patients with immunodeficiency or who were still receiving systemic steroid hormone therapy (prednisone > 10 mg/day or other equivalent drugs) or other forms of immunosuppressive therapy seven days prior to the first dose of neoadjuvant therapy in this study
(5) Clinical ascites or pleural effusion requiring therapeutic puncture or drainage
(6) Subjects with uncontrolled cardiac clinical symptoms or diseases, such as NYHA class 2 or more heart failure, unstable angina pectoris, myocardial infarction within 1 year, and clinically significant ventricular or ventricular arrhythmias requiring treatment or intervention.
(7) Abnormal coagulation function (PT>16s, APTT>43s, TT>21s, Fbg> 2G/L), bleeding tendency, or thrombolytic or anticoagulant treatment
(8) The subject presented (within 3 months) with esophageal varices, active gastric and duodenal ulcers, ulcerative colitis, portal hypertension, and other gastrointestinal diseases, active bleeding from unresected tumors, or other conditions that may cause gastrointestinal bleeding or perforation as determined by the investigator
(9) Past or present severe bleeding (bleeding >30 mL within 3 months), hemoptysis (fresh blood >5 mL within 4 weeks), or thromboembolism events (including stroke events and/or transient ischemic attack) within 12 months
(10) Patients with past or present objective evidence of pulmonary fibrosis, interstitial pneumonia, pneumoconiosis, radioactive pneumonia, drug-related pneumonia, or severely impaired lung function
(11) Patients with congenital or acquired immune deficiency, such as HIV infection, or active hepatitis (transaminase does not meet the inclusion criteria, hepatitis B reference: HBV DNA ≥ 10^4^/mL; Hepatitis C: HCV RNA ≥ 10^3^/mL); Chronic hepatitis B virus carriers, HBV DNA < 2000 IU/mL (<10^4^ copies/mL), must receive antiviral therapy during the trial to be enrolled
(12) Participation in other clinical trials
(13) A live vaccine was administered less than 4 weeks prior to study commencement or possibly during the study period.
(14) History of mental illness or psychiatric substance abuse.
(15) The subject cannot or does not agree to bear the cost of the self-paid portion of the examination and treatment, except for the clinical study drug, combined chemoradiotherapy, and serious AE associated with the clinical study drug.
(16) Other patients who were considered inappropriate for inclusion by the medical practitioner.

### Surgery

Surgery was performed four–six weeks after neoadjuvant therapy. Esophagectomy in patients with middle and lower ESCC required a total 2-field lymph node dissection, but the patients with upper ESCC required a complete 3-field lymph node dissection. The number of pathologically confirmed lymph nodes should be more than 15 in each enrolled patient. Thoracoscopic, open thoracotomy, and hybrid esophagectomies were all acceptable.

### Follow-Up

The primary endpoints will be analyzed three months after the surgical treatment of the last recruited patients. During the surveillance period, contrast-enhanced chest CT and cervical and abdominal ultrasound should be performed. Follow-up for recurrence or death will be conducted every three months for the first two years and every six months from the third to fifth years.

### Statistical Analysis

The time-to-event was estimated using the Kaplan–Meier method with the log-rank test to ascertain significance. Data were analyzed according to the intention-to-treat principle. The pCR rate in patients with esophageal cancer surgically resected after neoadjuvant radiotherapy and chemotherapy is approximately 35–45%. In our study, a more optimized preoperative neoadjuvant therapy mode was adopted, which was expected to achieve a better pCR rate. Therefore, our hypothesis was to increase the pCR rate by another 5–10%.

Thus, the trial required 80 eligible patients with complete histological response in the primary tumor with the use of a two-sided test with 0.80 statistical power and a significance level of 0.05.

The primary outcomes of the pCR rate, incidence of AEs, OS, ORR, MPR, and EFS were evaluated as secondary endpoints. All points were estimated by point and 95% confidence intervals were provided as representatives of the population. OS, 12- and 36-month survival, and median survival were estimated using Kaplan-Meier methods with 95% confidence intervals representing the population. Descriptive analysis was completed using statistics, including mean with standard deviation (SD), median with interquartile range (IQR), and frequency with percentage, and differences were considered significant at the 5% level (P < 0.05). Data were analyzed using SPSS software, version 23.0.

### Monitoring

The Good Clinical Practice (GCP) board and ethics committee of Sichuan Cancer Hospital evaluated patient safety, trial progress, and data integrity. The GCP reviewed the trial data every three months. The principal investigators (PI) were responsible for the design and performance of the study.

### Participating Institutions

Sichuan Cancer Hospital. Principal investigator: Lin Penguin, M.D.; Qifeng Wang, M.D.& Ph.D., PhD; Wenwu He, M.D., PhD.

### Funding, Registration, and Current Status

For this study, tislelizumab was offered free by the BeiGene Company, which was independent and without competing interests.

This study was registered at ClinicalTrials.gov in November 2021 (registration number NCT05189730). The protocol date and version identifier were version 1 (June 1th, 2021). Our study began with recruitment in July, 2021. The trial status was patient recruitment.

## Discussion

According to a national cancer center report, the incidence and mortality rates were 17.87/100000 and 13.68/100000, respectively, in China in 2015 (17). In addition, the incidence and mortality of esophageal cancer in men are higher than those in women, and they are higher in rural areas than in urban areas (17).

In Japan, most patients are diagnosed early due to early screening, and neoadjuvant chemotherapy is the standard treatment. However, in China, 80% of the patients diagnosed with ESCC are at a locally advanced stage. Although neoadjuvant chemoradiotherapy or chemotherapy is currently the standard treatment for resectable locally advanced ESCC, the survival rate of patients with ESCC is still limited due to high postoperative recurrence and metastasis rates (6, 7). To date, more than 50% of patients with ESCC show relapse within 1 year after surgery, even after neoadjuvant chemoradiotherapy (4, 18). If patients do not achieve pCR, the recurrence rate is even higher (8, 19). Therefore, improving the pCR rate and reducing recurrence and metastasis after neoadjuvant therapy are important in resectable locally advanced ESCC.

Keynotes 180 (12), 181 (10), and ATTRACTION-3 (11) studies showed that chemotherapy combined with immunotherapy achieved good outcomes in advanced ESCC, and immunotherapy has also been used for neoadjuvant therapy (13). However, these results did not indicate that chemotherapy plus immunotherapy could increase the pCR rate of neoadjuvant therapy for ESCC.

Previous basic research reports on immunotherapy and cancers showed that neoadjuvant chemoradiotherapy may upregulate PD-L1 expression (20). Therefore, we retrospectively compared the expression of PD-L1 before and after neoadjuvant chemoradiotherapy and neoadjuvant immunochemotherapy in our previous specimens, which showed that the expression of PD-L1 in both the groups was higher after treatment than that before treatment. In the chemoradiotherapy group, the expression of PD-L1 was elevated in 92.8% of patients, which was higher than that in the immunochemotherapy group, which showed a significantly increased IPS (P < 0.05).

In addition, another important study showed that the TNT model was found to increase the odds of pCR rate by 39% in neoadjuvant therapy for colorectal cancer (14), which is a promising strategy for locally advanced colorectal cancer, with superior rates of pCR compared with that of standard therapy (21–23).

To improve the pCR rate and reduce recurrence and metastasis rates in respectable locally advanced ESCC, we primarily conducted this exploratory study to answer cutting-edge questions.

## Conclusions

High recurrence and metastasis rates led to no significant survival benefit in neoadjuvant chemoradiotherapy plus surgery for resectable locally advanced ESCC. However, the survival benefit for patients in the pCR group after neoadjuvant chemoradiotherapy was significant. To improve the pCR rate of patients after neoadjuvant therapy and to reduce the risk of postoperative recurrence and metastasis rates, we conducted an exploratory study. This study provides a real survival benefit for ESCC patients.

This paper was prepared according to Standard Protocol Items: Recommendations for Intervention Trials (SPIRIT) (24).

## Data Availability Statement

The original contributions presented in the study are included in the article/supplementary material. Further inquiries can be directed to the corresponding authors.

## Ethics Statement

This study was approved by the ethics committtee of Sichuan Cancer Hospital, China. The ethics certificate number is SCCHEC-02-2021-056. The patients/participants provided their written informed consent to participate in this study.

## Author Contributions

WH, LP and QW designed the study. WH and CW drafted the manuscript. LW and GW registered the trial at Clinic Trial website, BL, YH, HL and XL helped the enrolled patients. QW and LP helped with the discussion and English copy editing, CHJ provided effective suggestion treatment and modification, DKY managed research data. All authors contributed to the article and approved the submitted version.

## Conflict of Interest

Author HC was employed by BeiGene (Beijing) Co., Ltd.

The remaining authors declare that the research was conducted in the absence of any commercial or financial relationships that could be construed as a potential conflict of interest.

## Publisher’s Note

All claims expressed in this article are solely those of the authors and do not necessarily represent those of their affiliated organizations, or those of the publisher, the editors and the reviewers. Any product that may be evaluated in this article, or claim that may be made by its manufacturer, is not guaranteed or endorsed by the publisher.
